# Head-shaking-induced nystagmus reflects dynamic vestibular compensation: A 2-year follow-up study

**DOI:** 10.3389/fneur.2022.949696

**Published:** 2022-09-20

**Authors:** Maja Striteska, Martin Valis, Viktor Chrobok, Oliver Profant, Luigi Califano, Jaroslav Syba, Katerina Trnkova, Jan Kremlacek, Martin Chovanec

**Affiliations:** ^1^Department of Otorhinolaryngology and Head and Neck Surgery, University Hospital Hradec Kralove, Charles University, Faculty of Medicine in Hradec Kralove, Hradec Kralove, Czechia; ^2^Department of Otorhinolaryngology, 3rd Faculty of Medicine, Charles University and University Hospital Kralovske Vinohrady, Prague, Czechia; ^3^Department of Neurology, University Hospital Hradec Kralove, Charles University, Faculty of Medicine in Hradec Kralove, Hradec Kralove, Czechia; ^4^Department of Auditory Neuroscience, Institute of Experimental Medicine of the Czech Academy of Sciences, Prague, Czechia; ^5^Audiology and Phoniatrics Department, San Pio Hospital Benevento, Benevento, Italy; ^6^Department of Biophysics, Charles University, Faculty of Medicine in Hradec Kralove, Hradec Kralove, Czechia; ^7^Department of Pathological Physiology, Charles University, Faculty of Medicine in Hradec Kralove, Hradec Kralove, Czechia

**Keywords:** head-shaking nystagmus, head-shaking test, head-shaking-induced nystagmus, vestibular compensation, follow-up study, velocity storage

## Abstract

**Purpose:**

We aimed to assess the ability of a head-shaking test (HST) to reflect vestibular compensation in patients after unilateral peripheral vestibular loss and to provide missing evidence and new insights into the features of head-shaking-induced nystagmus (HSN) over a 2-year follow-up.

**Background:**

HSN may occur after a prolonged sinusoidal oscillation of the head. HSN is frequently observed in subjects with vestibular function asymmetry; it usually beats toward the functionally intact or “stronger” ear and can be followed by a reversal of its direction.

**Study design:**

A prospective observational case-control study.

**Settings:**

A tertiary academic referral center.

**Methods:**

A total of 38 patients after acute unilateral vestibular loss (22 patients with vestibular neuronitis and 16 patients after vestibular neurectomy) and 28 healthy controls were followed for four consecutive visits over a 2-year period. A complex vestibular assessment was performed on all participants, which included spontaneous nystagmus (SPN), the caloric test, the head-shaking test (HST), the video head impulse test (vHIT), the Timed Up and Go (TUG) test, and the Dizziness Handicap Inventory (DHI) questionnaire. We established the criteria for the poorly compensated group to assess different compensatory behaviors and results.

**Results:**

We found a time-related decrease in HSN (ρ < −0.84, *p* < 0.001) after unilateral vestibular loss. After 2 years of follow-up, HSN intensity in compensated patients reached the level of the control group; TUG and DHI also improved to normal; however, the caloric and vHIT tests remained abnormal throughout all follow-ups, indicating a chronic vestibular deficit. Besides, poorly compensated patients had a well-detectable HSN throughout all follow-ups; TUG remained abnormal, and DHI showed at least a moderate deficit.

**Conclusions:**

Our study showed that, after a unilateral peripheral vestibular loss, the intensity of HSN decreased exponentially over time, reflecting an improvement in dynamic ability and self-perceived deficit. HSN tended to decline to the value of the control group once vestibular compensation was satisfactory and sufficient for a patient's everyday life. In contrast, well-detectable HSN in poorly compensated patients with insufficient clinical recovery confirmed the potential of HSN to reflect and distinguish between adequate and insufficient dynamic compensation. HSN could serve as an objective indicator of stable unilateral vestibular loss.

## Introduction

Head-shaking-induced nystagmus (HSN) was first described by Bárány (1); the first formal description was given by Vogel (2) and the test was standardized by Kamei et al. (3). HSN is a jerk nystagmus that may occur after a prolonged sinusoidal oscillation of the head and lasts at least a few seconds (4). HSN in the horizontal or vertical plane is abnormal. In subjects with vestibular function asymmetry, HSN is frequently observed, usually beating toward the functionally intact or “stronger” ear, and may be followed by a reversal of its direction (5, 6). The head-shaking test (HST) is an easy-to-perform test and can be performed as a low-cost bedside test with minimal equipment (Frenzel goggles) or can be assisted with video oculography (VOG) for precise evaluation of the slow phase of nystagmus.

Head-shaking-induced nystagmus was described in the literature corresponding to vestibular function asymmetry (7–9) and might be associated with the degree of functional deficit (10). In contrast, other studies found varying degrees of sensitivity and specificity in identifying such an asymmetry (11, 12). The discrepancy between the conclusions raises the question of whether HSN may reflect dynamic compensation rather than vestibular asymmetry.

Our study aimed to provide long-term missing evidence and new insights into the features of HSN in patients with unilateral vestibular loss (UVL) over a 2-year follow-up period. We tested the feasibility of HST to reflect dynamic vestibular compensation in UVL. To date, we have not found any literature on the long-term properties of HSN that evaluates HSN as a possible indicator of individual dynamic compensation.

## Methods

### Participants

We used data from 66 participants: 28 healthy volunteers and 38 patients (22 patients with vestibular neuronitis and 16 patients after vestibular neurectomy). Subsequently, we divided participants into four groups according to their different vestibular behaviors, results, and complaints.

### Vestibular neuronitis, surgery, and control groups

The inclusion criteria for the vestibular neuronitis group included a history of the first vertigo attack and a confirmed acute UVL without any neurological or cochlear deficit. According to the HINTS plus protocol (13–15) and normal magnetic resonance imaging (MRI) results, peripheral vestibular deficit was proven. For the surgery group, subjects with MRI-confirmed vestibular schwannomas [Koos classifications 1–2 (13)] with normal or near-normal vestibular function before the surgery were included. The inclusion criteria for the control group included no evidence of any balance problems currently or in the past, no history of vestibular disease, and normal hearing thresholds. We examined participants from January 2018 to February 2022.

### Poorly compensated group

To assess the features of HSN during vestibular compensation, we defined the criteria for poorly compensated patients as those who had a unilateral vestibular deficit [>26% unilateral weakness (UW) by a bithermal caloric test] and who had at least three of the following criteria: complaints of blurred vision (subjectively often described as slow or lazy eyes) during daily life head turns (e.g., turning the head to the left and right before crossing the street) or dizziness, defined as a total score higher than 16 on the Dizziness Handicap Inventory (DHI) (14), having a gait disturbance defined as a timed up and go (TUG) test score more than 10 s (or in need of assistance), or having spontaneous nystagmus (SPN). According to our criteria, we planned to establish a poorly compensated group after V4 was completed because there is no test to confirm sufficient and finished dynamic compensation. Six patients fulfilled the criteria during all follow-ups, and *post-hoc* formed the poorly-compensated group.

*Post-hoc*, we established four groups for comparison: the neuronitis and surgery groups (compensated patients), the poorly compensated group, and the control group. We analyzed 20 subjects with vestibular neuronitis (neuronitis group, five women, 15 men, mean age 45 years), 12 patients with vestibular schwannoma after unilateral vestibular neurectomy (surgery group, two women, 10 men, mean age 51), and six subjects who were poorly compensated from both groups (poorly compensated group, two from neuronitis and four from the surgery group, five women and one man, mean age 49 years). The control group consisted of 28 volunteers (15 women, 13 men, mean age 48 years).

### Settings

We examined all subjects at a tertiary referral center, University Hospital.

### Measurements

We measured average slow phase velocity (aSPV) [°/s] of spontaneous nystagmus (SPN) and head-shaking-induced nystagmus (HSN), unilateral weakness (UW) during the caloric test, and video head impulse test (vHIT) gains.

### Devices used for a study

VOG VisualEyes™ 525 (Interacoustics, Denmark) and VHIT EyeSeeCam (Interacoustics, Denmark) were used to perform a complex vestibular examination on all participants.

### SPN recording method

The patient was asked to sit upright, and visual fixation was denied. The tracing was recorded for 40 s (sitting position, head still, and goggle closure). If any nystagmus occurred, the VOG software measured its slow phase component.

### VOG-assisted bithermal air caloric test procedure

The patient was asked to lie in a caloric position, and each ear was irrigated with warm (50°C) and cold (24°C) air for 60 s and nystagmic response was recorded for 120 s.

### HST procedure

The patient was asked to sit upright, and visual fixation was denied. Eye movements were observed for 10 s to obtain a baseline. The examiner moved the patient's head (pitched forward 30°) briskly to the left and the right, aiming for a frequency of ~2 Hz and a head displacement of roughly 40–60°, 20 cycles (duration of 10 s), and then stopped abruptly. VOG was recorded for 120 s; induced nystagmus was evaluated. If there were more than two repetitive nystagmus beats after a headshake, they were analyzed.

### VHIT procedure

The patient was asked to sit upright, with visual fixation of a spot at a distance of approximately 1 m, unpredictable and passive head turns, a peak head velocity of between 150° and 250°/s, and the amplitude of a head turn being 10–20°.

### Questionnaires

The subjective functional status of participants was assessed using the DHI questionnaire, which represents the functional, emotional, and physical aspects of subjectively reported disability (14).

### Timed up and go test

The Timed Up and Go test measures functional mobility to estimate the risk of falling and the ability to maintain balance while walking. The patient was asked to sit in a chair; after the examiner said “go,” the timer started, and the patient got up from the chair, walked a distance of 3 m, turned and walked back to the chair, and sat down again and the timer stopped. We also evaluated the need for assistance. One limitation of the TUG test was the subjective connotation of the “normal walking speed.” Some could interpret this as a brisk walk, while others interpreted it as a leisurely pace.

### Scheduled follow-ups

We scheduled four examinations: the first (V1) within the 1st week after unilateral vestibular loss (UVL), the second (V2) after 4–6 months, the third (V3) after 12 months, and the final (V4) after 24 months.

### Variables, bias, study size

Our study used continuous quantitative variables (aSPV of SPN and HST in degrees/s, caloric weakness in %, vHIT gains). To evaluate the potential of HSN to reflect vestibular compensation after UVL, we had to exclude potential sources of bias. First, compensation for previous vestibular loss, which could be present in the schwannoma group before the surgery, was possibly and might have already started or even completed. A longer compensation period could give false results during the scheduled 2-year period. Second, normalization of vestibular function (functional recovery) at follow-up in the neuronitis group would not assess a compensation process. Finally, differences in behavior exist in the poorly compensated group.

To minimize bias, we first established two study groups in which normal or near-normal vestibular function was expected before unilateral vestibular loss (measured in the surgery group and expected in neuronitis without a history of imbalance). We excluded patients who had abnormal vHIT gains and significant caloric weakness present before surgery (the surgery group consisted of small Koos 1 or 2 tumors).

Second, we *post-hoc* excluded neuronitis patients with normalized vestibular function (normalized caloric test) and performed a *post-hoc* analysis of patients with significant vestibular loss (caloric weakness >26%) during all scheduled follow-ups.

Finally, we *post-hoc* established a poorly compensated group according to the defined criteria to reflect differences in behavior between poorly and well-compensated patients after UVL.

### Statistical analysis

As part of the descriptive statistics, we evaluated the normality of the data distribution using the Anderson–Darling test for the average velocities of the slow phase of SPN, HSN, caloric weakness, and gains from the vHIT regression analysis in all groups. Because some continuous variables did not show a normal distribution, we reported medians and lower and upper quartiles for all continuous variables. To compare patient data to control group values, we used an unpaired two-tailed *t*-test in case of confirming the normality of the data distribution. Otherwise, a non-parametric two-tailed Wilcoxon rank-sum test was used.

We used linear regression analysis to statistically evaluate the evolution of examination results over time. Because the exploratory analysis showed exponential dependencies, we log-transformed time. We then calculated the slope of the regression line, hereafter referred to as the trend, for each patient. We tested the set of individual trends using the one-sample *t*-test or the Wilcoxon test for significant differences from 0.

The relationship between the examination methods was assessed using the correlation analysis of individual trends. The individual trend was calculated using linear regression, and, as the data showed an exponential dependency, time was logarithmized. The relationship between the methods was calculated using Pearson's test in the case of a normal distribution and Spearman's test in the absence of a normal distribution. A significant positive correlation between the methods indicates that faster improvement in one method leads to a faster improvement in the other.

To compare the sensitivity between the examinations, we contrasted the measured values with the cutoffs as follows. The abnormal cutoffs for HSN (2.03°/s) and SPN (1.70°/s) were determined as 97.5% of our control group, 26% UW for the caloric test, and a gain of 0.78 for vHIT (15). We assessed sensitivity separately for each visit (V1–V4). Individual tests were not corrected for multiple comparisons because this would increase the likelihood of false negative results. R-project software was used for statistical processing (R Development Core Team 2022) (10).

### Standard protocol approvals, registrations, and patient consents

Before including a subject in this study, we received written informed consent signed by a volunteer/patient.

## Results

**Figure 1:**
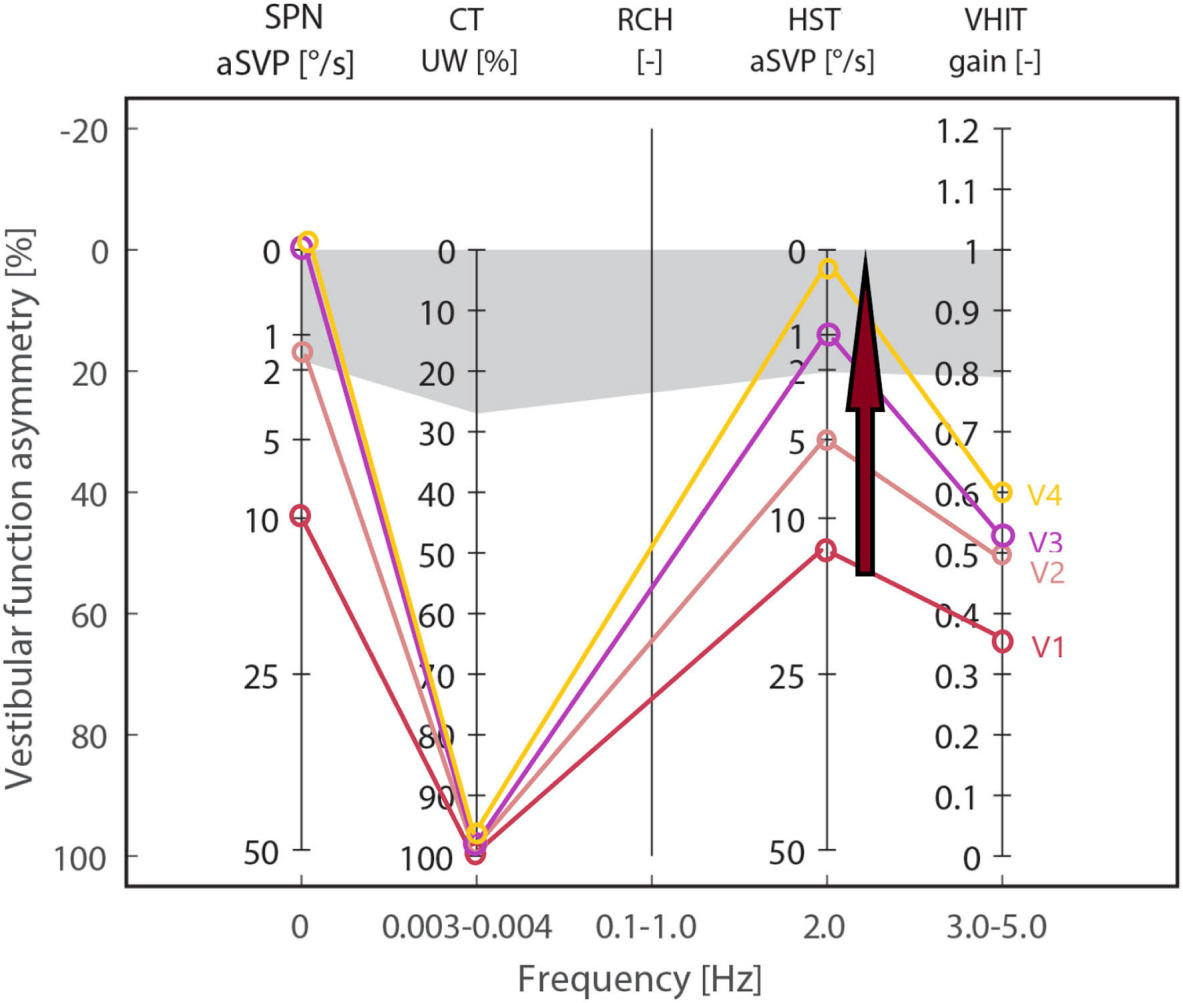
Estimated Vestibulogram (EVEST) for the neuronitis group, affected side: Median values from each visit are depicted to visualize an HSN-intensity decreasing trend (red arrow) during 2 years of follow-ups (visits V1–V4) from the abnormal to control group level. The same trend is observed in SPN till normalization. HST, head-shaking test; SPN, spontaneous nystagmus; vHIT, video head impulse test; CT, caloric test; gray zone corresponds to abnormal cut-offs calculated from a control group.

We attached the results of each group during all visits and reported the test trends during the follow-ups and the significance of the intergroup difference (between the results of each group and the control group) in Table 1. To visualize the trend of each test during a 2-year follow-up, we depicted the median values of the vestibular neuronitis group on an Estimated Vestibulogram (EVEST) (16) in Figure 1.

**Table 1:** Groups results: For the vestibular tests the medians and lower and upper quartiles are shown (HST, head-shaking test; SPN, spontaneous nystagmus; vHITfe(af), video head impulse test on affected (fellow) side; CT, caloric test; TUG, timed up and go test; DHI, dizziness handicap inventory).

		**Visit 1**	**Visit 2**	**Visit 3**	**Visit 4**	**Trend [ln(months)]**	***p***
Neuronitis group (*n* = 20)	HST [°/s]	15* (9.75; 19.25)	5* (3; 6.5)	1* (1; 2)	0.25 (0; 1.925)	−4.848 (−5.65; −2.76)	9.19e-09
	SPN [°/s]	9.5* (6.75; 13.5)	1.6* (0; 2.5)	0 (0; 1)	0 (0; 0)	−3.25 (−4.38; −2.15)	4.86e-07
	vHITfe [–]	0.85 (0.78; 0.91)	0.90 (0.82; 0.98)	0.87 (0.8; 0.94)	0.90 (0.86; 1.02)	0.022 (−0.007; 0.055)	0.035
	vHITaf [–]	0.36* (0.298; 0.42)	0.5* (0.38; 0.67)	0.53* (0.45; 0.70)	0.6* (0.56; 0.69)	0.077 (0.043; 0.11)	5.41e-6
	CT [%]	100* (100; 100)	100* (89; 100)	100* (57; 100)	100* (56; 100)	0.0 (−13.7; 0.0)	0.003
	TUG [s]	20* (18; 23)	8.0 (8.0; 9.0)	8.0 (8.0; 9.0)	8.0 (7.0; 8.75)	−3.82 (−4.7; −3.7)	1.42e-08
	DHI [–]	86* (80; 90)	44* (38; 48)	10* (8; 12)	8* (6; 8)	−26.28 (−27.4; −24.7)	2.91e-20
Surgery group (*n* = 12)	HST [°/s]	12.5* (11.5; 15.5)	7* (5.25; 8.75)	2* (1; 6)	0.25 (0; 2)	−3.55 (−4.58; −2.5)	6.39e-06
	SPN [°/s]	12.5* (7.5; 14.75)	2 (1; 2.75)	0 (0; 1)	0 (0; 0.125)	−4.07 (−5.34; −2.28)	2.09e-05
	vHITfe [–]	0.79 (0.73; 0.95)	0.865 (0.815; 0.937)	0.83 (0.79; 0.9)	0.835 (0.8; 1)	0.014 (−0.033; 0.049)	0.334
	vHITaf [–]	0.35* (0.23; 0.39)	0.36 (0.26; 0.46)	0.37 (0.24; 0.455)	0.435 (0.32; 0.49)	0.017 (−0.006; 0.062)	0.118
	CT [%]	100* (100; 100)	100 (100; 100)	100 (100; 100)	100 (100; 100)	0.0 (0.0; 0.0)	0.02
	TUG [s]	20* (14; 20.25)	10* (10; 10.75)	9* (8.75; 9)	9* (9)	−3.4 (−4.58; −1.69)	0.0004
	DHI [–]	88* (80; 90)	40* (36; 47)	14* (11.5; 16)	8 (6.5; 10)	−25.68 (−27.9; −24.1)	3.14e-12
**Poorly-compensated (*****n*** **=** **6)**	HST [°/s]	15* (12; 16.5)	6.5* (4.5; 8.5)	6* (2; 7)	3.5* (2; 5)	−3.002 (−3.98; −2.83)	0.031
	SPN [°/s]	11.5* (9.25; 14.5)	3* (2; 3)	2* (1.75; 2.25)	1* (1)	−3.191 (−4.14; −2.58)	0.031
	vHITfe [–]	0.79 (0.74; 0.94)	0.82 (0.78; 0.92)	0.8 (0.79; 0.9)	0.83 (0.78; 0.86)	−0.004 (−0.025; 0.004)	0.843
	vHITaf [–]	0.37* (0.34; 0.39)	0.38* (0.35; 0.42)	0.4* (0.38; 0.48)	0.41* (0.38; 0.57)	0.018 (0.011; 0.031)	0.031
	CT [%]	100* (100; 100)	100* (100; 100)	100* (100; 100)	100* (100; 100)	0 (0; 0)	1.000
	TUG [s]	22* (20; 23)	11* (11)	12* (11.75; 15.5)	11.5* (11; 12.75)	−3.17 (−3.65; −1.54)	0.0935
	DHI [–]	85* (80; 90)	54* (50; 60)	24* (20; 30)	17* (14.5; 34.5)	−17.68 (−22.49; −8.97)	0.031

Descriptive characteristics are listed for each visit (visits 1–4). The trend column shows an estimate of the linear evolution of the tests over time. Because the data showed an exponential pattern in time, it was logarithmized. Trend values are shown with 95% confidence intervals. The column labeled p indicates the statistical significance of the hypothesis that the trend is different from zero. The significant difference between the group results and the control group was assigned with a (^*^).

For comparison, the results of the control group were: HST 0.5 (0; 1.05), SPN 0 (0; 0.625), vHIT 0.9 (0.858; 0.935) and 0.89 (0.815; 0.927), caloric weakness 12 (8.5; 15.5), TUG 8 (7; 9), and DHI 0 (0; 0).

### Comparison between groups and controls

#### SPN

When SPN in both the neuronitis and surgery groups was compared to the control group, there were significant differences in V1 and V2, and there were no intergroup differences in V3 and V4 between the groups. In contrast, there was a significant difference between the poorly compensated and control groups in all examinations (for statistical significance, see Table 1). The results showed a reduction of SPN to normal within the 1st year in the neuronitis and surgery groups, reflecting a finished static VOR compensation after UVL in contrast to the poorly compensated group who showed detectable SPN even 2 years after UVL.

#### HSN

When HSN in both the neuronitis and surgery groups was compared to the control group, there were significant differences at V1–V3, while there were no intergroup differences at V4. In contrast, there was a significant difference during all visits in the poorly compensated group. The results showed a decreasing trend of HSN intensity in the neuronitis and surgery groups in contrast to the poorly compensated individuals who showed a well-detectable HSN in all follow-ups.

#### Caloric test

When caloric weakness in all groups was compared to the control group, there were significant differences in visits V1–V4, indicating a vestibular deficit at all follow-ups.

#### VHIT affected

When the vHIT-affected side in all groups was compared to the control group, there were significant differences in visits V1–V4, showing a detectable vestibular deficit at all follow-ups.

#### TUG

There was a significant difference between the neuronitis and control groups in V1, but no intergroup difference in the remaining follow-ups, reflecting sufficient gait control. There was a significant difference between the surgery and control groups at each visit. However, the surgery patients had TUG at V2–V4 below the abnormal cutoff (10 s), indicating sufficient gait control. There was a significant difference between poorly compensated and control groups in all examinations, while the poorly compensated group showed TUG above the abnormal cutoff in all visits.

#### DHI

When DHI in all groups was compared to the control group, there were significant differences between the groups in all examinations. In contrast to the poorly compensated group, the DHI score median in the neuronitis and surgery groups was within a normal range (below 16 points) at V3 and V4. The poorly compensated group reported a mild self-perceived handicap (16–34 points) even at V3 and V4.

#### Summary

The results showed that SPN decreased to a control group level after 1 year, while HSN decreased after 2 years in the majority of compensated patients. HSN was more intense or similar in intensity to SPN at V1 and was significantly more intense at V2 (in all groups). In contrast to the poorly compensated group (median 2), SPN disappeared at V3 in compensated groups (median 0). HSN was still present at V3 (median 1 in the neuronitis group and 2 in the surgery group, 6 in the poorly compensated group). After 2 years (V4), HSN intensity in the compensated groups also reached the control group's result (median 0.25 in the neuronitis group and 0.3 in the surgery group) in contrast to the poorly compensated group (median 3.5).

In contrast, despite a slight improvement in the neuronitis group, the caloric and vHIT tests remained abnormal during the followed period, indicating a chronic vestibular deficit.

We used a TUG (the ability to maintain balance while walking) and the DHI questionnaire (measures the self-perceived handicap) to assess, to assess the dynamic vestibular function and the impact of dizziness on daily life, respectively. TUG and DHI improved to normal in the compensated neuronitis and surgery groups at follow-ups but not in the poorly compensated group. The poorly compensated group showed significant SPN and HSN intensities, abnormal TUG, and higher DHI scores during/ at all follow-ups.

### Trend analysis

**Figure 2:**
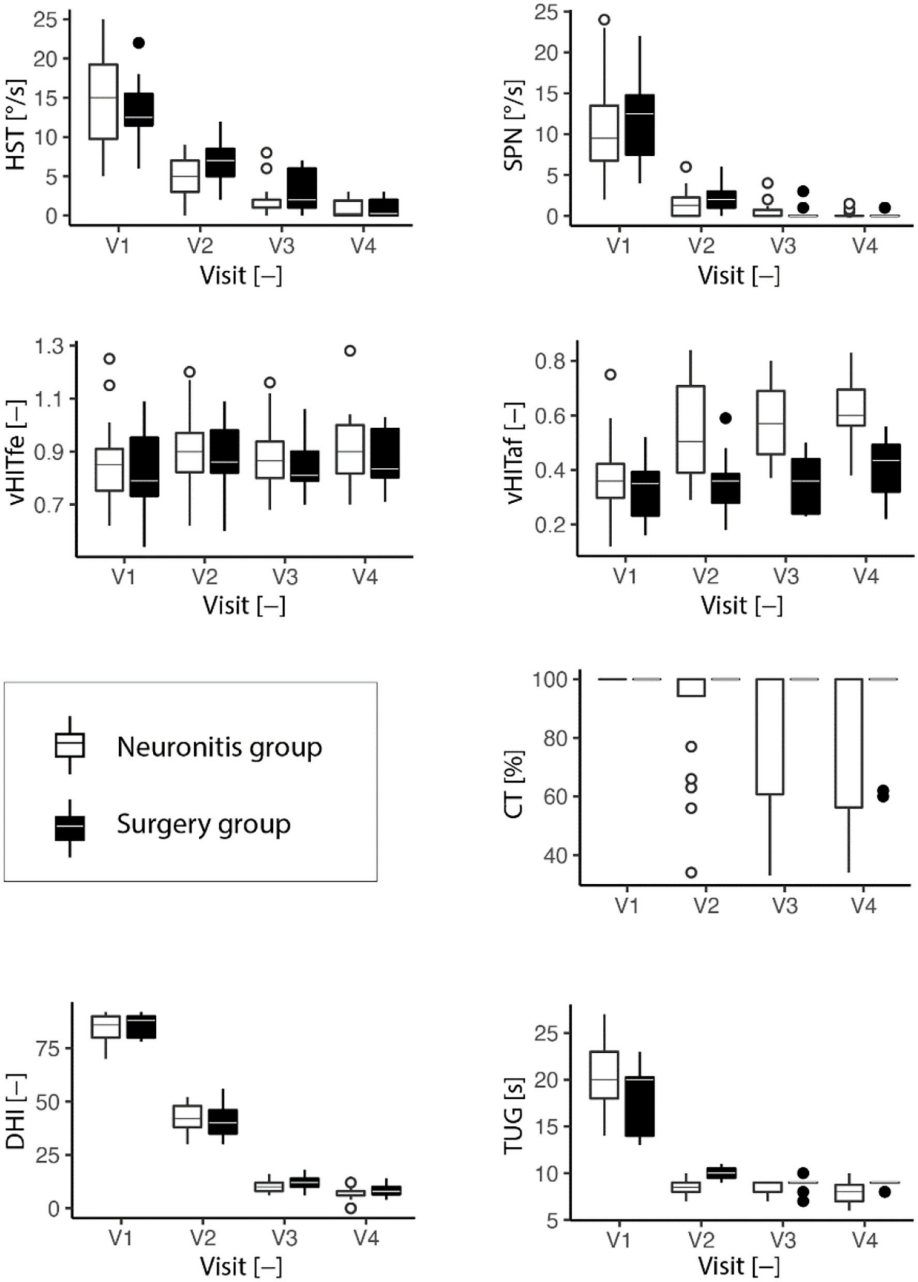
Development of the vestibular tests (HST, head-shaking test; SPN, spontaneous nystagmus; vHITfe(af), video head impulse test on the affected (fellow) side; CT, caloric test; TUG, timed up and go test; DHI, dizziness handicap inventory) over four visits (the last visit). Descriptive characteristics are listed for each visit (2 years). The box plots are depicted. In the boxplots, the bottom line of the box represents the first quartile, second (middle of the box) median, third (top of the box) quartile, and the whiskers extend to the most extreme data point, but no more than one and half of the interquartile range.

The result trends from all vestibular tests are presented in Table 1 and visualized in Figures 1, 2 for the neuronitis group and the neuronitis and surgery groups, respectively.

### Correlation analysis

#### Time correlated to each test

We did a correlation analysis between time and each test for the neuronitis and surgery groups (as only a few results were within the poorly compensated group). We found strong and highly significant negative correlations between time and HSN intensity (Spearman's ρ < −0.84, *p* < 0.001), time and SPN (ρ < −0.80, *p* < 0.001), time and TUG (ρ < −0.67, *p* < 0.001), and time and DHI (ρ < −0.94, *p* < 0.001) in both the groups. The results confirmed the time-related improvement of these tests.

We found a weak relation between time and the caloric test in the neuronitis (ρ = −0.34, *p* = 0.005) and surgery groups (ρ = −0.27, *p* < 0.047), as well as a weak positive relation between time and vHIT-affected side in the neuronitis (ρ = 0.52, *p* < 0.001) and surgery groups (ρ = 0.28, *p* = 0.040). Both the groups showed no significant relationship between time and vHIT on the fellow side (ρ <0.26, *p* > 0.137). The correlation analysis reflected an almost stable caloric weakness (indicating the presence of vestibular loss/asymmetry) or just a slight improvement in vHIT (but still abnormal) during follow-up.

#### HSN correlations to the remaining tests

To assess the similar or different trends for improvement, we performed a correlation analysis between HSN and the remaining tests for the neuronitis and surgery groups. We did not perform a correlation analysis for the poorly compensated group according to a small group size.

Head-shaking-induced nystagmus vs. SPN (*r* = 0.877; *p* < 0.001) were significantly correlated with each other. A significant positive correlation indicates a simultaneous individual change in the results of each examination over time, and a faster improvement in one method leads to a faster improvement in the other method.

Head-shaking-induced nystagmus vs. vHIT affected side (*r* = −0.14; *p* = 0.44) as well as HSN vs. caloric weakness (ρ = −0.07; *p* = 0.67) were not correlated because HSN had a decreasing trend, whereas vHIT and caloric test had stable trends.

Interestingly, HST vs. DHI (*r* = 0.192; *p* = 0.28) and TUG (*r* = 0.055; *p* = 0.82) were not correlated despite the decreasing (improving) trends. The explanation could be a rapid improvement in TUG from V1 to V2, followed by almost stable results, similar to the improvement of V3–V4 in DHI. In contrast, the intensity of HSN decreased across all visits.

### Specificity and sensitivity

We contrasted the measured values to the following cutoffs to compare the sensitivity and specificity (to identify vestibular loss) between the different vestibular tests. Abnormal cutoffs for HSN (2.03°/s) and SPN (1.70°/s) were determined to be 97.5% of our control group (as continuous variables did not show a normal distribution); the abnormal cutoff for the caloric test was used as 26% of UW and for vHIT was a gain of 0.78 (15).

The specificity for identifying controls was 94% for HST, 93% for SPN, 94% for caloric weakness, and 75% for vHIT.

To assess a trend to identify a vestibular loss (vestibular asymmetry), we calculated the sensitivity for each visit (V1–V4) separately. In the neuronitis group, the sensitivity of HST was 100% at V1, 79% at V2, 24% at V3, and 6% at V4. The sensitivity of SPN was 100% at V1, 47% at V2, 12% at V3, and 0% at V4. The sensitivity of vHIT was 100% at V1, 84% at V2, 94% at V3, and 83% at V4. The caloric test sensitivity was 100% on each session.

In the surgery group, the sensitivity of HST was 100% at V1, 93% at V2, 42% at V3, and 20% at V4. The sensitivity of SPN was 100% at V1, 57% at V2, 17% at V3, and 0% at V4. The sensitivity of vHIT and caloric weakness was 100% at all sessions.

In the poorly compensated group, the sensitivity of HST was 100% at V1 and V2, 66% at V3, and 50% at V4. The SPN was 100% at V1 and V2, 83% at V3, and 17% at V4 (a weak SPN of at least one aSPV was present in all poorly compensated groups).

To summarize, the caloric test and vHIT showed almost stable sensitivity to detect vestibular loss, whereas SPN and HST demonstrated a strongly decreasing ability to detect asymmetry, particularly in compensated patients.

## Discussion

### Theoretical explanation of HST

Several mechanisms have been proposed to explain HSN (5, 9). The etiology of HSN is thought to be related to both Ewald's second law and an asymmetry in the central velocity storage mechanism (17). In healthy subjects, each head turn is immediately opposed by a contralateral head turn during a head shake, resulting in a balanced ratio of excitation/inhibition from both sides.

In vestibular loss, there is a need to reestablish tonic and phasic vestibular function. Evidence suggests that second-order vestibular neurons tend to modulate their neuronal resting discharge and reach prelesion levels (16, 18–20), placing the site of neural rearrangement in commissural pathways (21), leading to clinical improvement and static compensation. Due to inhibitory saturation, the centrally restored pacemaker discharge is insufficient to restore the whole ipsilesional dynamic range (22). Therefore, during HST, head turn toward the healthy side is opposed during ipsilesional head turn only by inhibitory cutoff from the healthy side. Excitation is more effective than inhibition as a vestibular stimulus. During 20 cycles of HST, non-linearity arises and may charge the velocity storage mechanism in an asymmetric manner. When the head abruptly stops, HSN will result from the discharge of the asymmetrically charged velocity storage mechanism.

Studies suggest that, when lower frequency sinusoidal stimuli are used, the performance of the VOR often recovers over time, and asymmetries are only noted at higher rotational velocities with increasing head velocity (23) as a result of linear and nonlinear VOR pathways (24, 25).

### Clinical utility of HST

Head-shaking test was extensively tested for its sensitivity and specificity in identifying different vestibular diagnoses and in patients with peripheral and central vestibular lesions (3, 4). The presence of HSN in peripheral vestibular lesions varies between 34% (26), 40% (27), 90% (28), and 100% (17) and was also reported in benign positional paroxysmal vertigo (29, 30), in central disorders of 23% and was found to be present in 10–14% of healthy controls (3, 26), in 74% of dizzy patients (3), and 15% dizzy but normal patients with electronystagmography (31). The well-established literature review by Burgio (32) reported that HST is neither sensitive nor specific enough to be used as a screening test for vestibular loss. Another study found a closer connection between HSN and poorly compensated UVL than functional asymmetry (33).

To conclude, research results appear to be inconsistent. Some studies used active head movements, while others used passive head movements; some used scleral search coil, ENG, or VNG, while others used only Frenzel goggles. The study size, patient inclusion criteria, and the HST method also differed. We found different HST amplitudes (half a distance) in the literature, varying from 15 to 45° (6, 8, 10, 17, 34), resulting in different velocities (120–360°/s).

The authors attempted to explain discrepancies between the studies and suggest that some may arise from partial UVL, which may not have sufficient asymmetry, to elicit HSN, or that the central velocity storage may be reduced or lost after UVL. Others point out that HST evaluates a wider frequency range than the standard caloric or rotary chair stimulation (31).

We support the suggestion that HSN should always be interpreted in relation to other tests, such as SPN, VHIT, and a side of caloric weakness (35).

As the evidence appears inconclusive, the role of HST in clinical practice is still unclear.

### Our results

To summarize our results, HSN was not related to vestibular asymmetry (loss) at all follow-ups, but it did show a strong time-related intensity decrease, reflecting an improvement in dynamic ability and a decrease in self-perceived handicap. The decreasing trend in HSN sensitivity to identify vestibular loss also supports the ability of HSN to reflect dynamic vestibular compensation after UVL. Similar results were revealed by Angeli et al. (33), suggesting that HSN had a stronger correlation with poorly compensated unilateral peripheral loss than functional asymmetry.

According to evidence, ~20% of patients with chronic stable UVL continued to experience chronic postural imbalance (the syndrome of chronic vestibular insufficiency) (36). We had a similar report of 15% poorly compensated patients, showing a well-detectable HSN and SPN as well as a higher TUG and DHI score at all follow-ups.

We provide the first long-term evidence of the features of HSN, showing the ability of HSN to reflect dynamic vestibular compensation and decreasing sensitivity to identify chronic and stable vestibular asymmetry. We found no theoretical explanation for the decreasing trend in the sensitivity of an HST to identify vestibular loss (high sensitivity in the 1st year after UVL and low sensitivity in 2 years). We found no literature based on animal electrophysiological experiments on chronic adaptive changes of the VOR explaining long-term (years) characteristics in velocity storage and on long-term dynamic changes of the VOR more than a few months after UVL. We hypothesize that the sensitivity of HST to identify vestibular asymmetry could be decreased by individual adaptive changes in velocity storage, including the use of non-linear/linear pathways to compensate for dynamic asymmetry during a long-term compensation process.

### Strength of this study

Despite the small sample size (due to strict inclusion/exclusion criteria such as “no-allowed-recovery” and “no-allowed-prior-deficit”), the power size of the main message (time-related decrease in HST intensity) was strong (for the neuronitis group: Spearman's ρ = −0.8436362; *p* = 3.228042e-19, two-tailed test: effect size and confidence limits, *d* = −3.14 [−4.04 −2.24]), (for schwannoma group ρ = −0.8912293; *p* = 3.819181e−19, two-tailed test: effect size and confidence limits, *d* = −3.93 [−5.13 −2.73]) and the power of the study with aforementioned effect was = 1.

## Conclusions

Our study showed that, after UVL, HSN intensity decreases exponentially with time, reflecting an improvement in dynamic ability and self-perceived deficit in most patients. Once vestibular compensation was satisfactory and sufficient for a patient's daily life, HSN tended to decline to a control group's value. In contrast, poorly compensated patients with insufficient clinical recovery showed a well-detectable and more intense HSN during all follow-ups. HSN could serve as an objective vestibular indicator of individual dynamic compensation. However, these findings should always be interpreted with respect to other results, such as SPN and a side of caloric or vHIT deficit.

## Data availability statement

The anonymized data supporting the conclusions of this article will be shared by request to any qualified investigator.

## Ethics statement

The study was performed according to the ethical standards of the Declaration of Helsinki and following procedure approval of the Ethics Committee of the University Hospital Hradec Kralove (Reference number 202012 P03).

## Author contributions

MS and JK conducted the study. MS and KT did measurements. MS wrote the manuscript and JK provided statistical analysis. All other authors provided critical revisions to the draft, read, and approved the final manuscript.

## Funding

This work was funded by the Research Project of the Charles University, the Cooperatio Program, research area SURG, Faculty of Medicine in Hradec Kralove and 3rd Faculty of Medicine.

## Conflict of interest

The authors declare that the research was conducted in the absence of any commercial or financial relationships that could be construed as a potential conflict of interest.

## Publisher's note

All claims expressed in this article are solely those of the authors and do not necessarily represent those of their affiliated organizations, or those of the publisher, the editors and the reviewers. Any product that may be evaluated in this article, or claim that may be made by its manufacturer, is not guaranteed or endorsed by the publisher.

## Abbreviations

aSPV: average slow phase velocity
HST: head-shaking test
hVOR: horizontal vestibuloocular reflex
L-SCC: lateral semi-circular canal
SPN: spontaneous nystagmus
UW: unilateral weakness
VOR: vestibuloocular reflex
VOG: videooculography
VHIT: video head impulse test
DHI: dizziness handicap inventory; Questionnaire.
